# Characterization of a *Trametes versicolor* aflatoxin B1-degrading enzyme (TV-AFB1D) and its application in the AFB1 degradation of contaminated rice *in situ*

**DOI:** 10.3389/fmicb.2022.960882

**Published:** 2022-09-14

**Authors:** Peizhou Yang, Wei Xiao, Shuhua Lu, Shuying Jiang, Suwei Jiang, Jianchao Chen, Wenjing Wu, Zhi Zheng, Shaotong Jiang

**Affiliations:** ^1^College of Food and Biological Engineering, Anhui Key Laboratory of Intensive Processing of Agriculture Products, Hefei University of Technology, Hefei, China; ^2^Department of Biological, Food and Environment Engineering, Hefei University, Hefei, China

**Keywords:** *Saccharomyces cerevisiae*, *Escherichia coli* BL21 (DE3), aflatoxin B_1_, aflatoxin degrading enzyme, enzymatic characteristics, CRISPR-Cas9

## Abstract

Aflatoxin B_1_ (AFB_1_) contaminates rice during harvest or storage and causes a considerable risk to human and animal health. In this study, *Trametes versicolor* AFB_1_–degrading enzyme (TV–AFB_1_D) gene recombinantly expressed in engineered *E. coli* BL21 (DE3) and *Saccharomyces cerevisiae*. The TV–AFB_1_D enzymatic characteristics and AFB_1_ degradation efficiency in contaminated rice were investigated. Results showed that the size of recombinant TV-AFB_1_D expressing in *E. coli* BL21 (DE3) and *S. cerevisiae* was appropriately 77 KDa. The kinetic equation of TV-AFB_1_D was *y* = 0.01671*x* + 1.80756 (*R*^2^ = 0.994, *K_m_* = 9.24 mM, and *V_max_* = 553.23 mM/min). The *Kcat* and *Kcat*/*K_m_* values of TV-AFB_1_D were 0.07392 (s^−1^) and 8 M^−1^ s^−1^, respectively. The AFB_1_ concentration of contaminated rice decreased from 100 μg/ml to 32.6 μg/ml after treatment at 32°C for 5 h under the catabolism of TV-AFB_1_D. *S. cerevisiae* engineered strains carrying aldehyde oxidase 1 (*AOX1*) and *Cauliflower mosaic virus* 35 S (*CaMV* 35 S) promoters caused the residual AFB_1_ contents, respectively, decreased to 3.4 and 2.9 μg/g from the initial AFB_1_ content of 7.4 μg/g after 24 h of fermentation using AFB_1_-contaminated rice as substrate. The AFB_1_ degradation rates of *S. cerevisiae* engineered strains carrying *AOX1* and *CaMV* promoters were 54 and 61%, respectively. Engineered *S. cerevisiae* strains integrated with TV-AFB_1_D expression cassettes were developed to simultaneously degrade AFB_1_ and produce ethanol using AFB_1_-contaminated rice as substrate. Thus, TV-AFB_1_D has significant application potential in the AFB_1_ decomposition from contaminated agricultural products.

## Introduction

Aflatoxins (AFs) are a group of secondary metabolites produced by *Aspergillus flavus* and *Aspergillus parasiticus* that have highly teratogenic, carcinogenic, and mutagenic properties (Lee et al., 2022). AF contamination has posed a serious risk (Yang et al., 2020) and huge economic losses to food safety (Garcia-Cela et al., 2019), as well as human and animal health (Javed et al., 2021). AFs are classified as AFB_1_, AFB_2_, AFG_1_, AFG_2_, AFM_1_, AFM_2_, AFP_1_, and AFQ_1_. AFB_1_ is the most toxic with acute toxicity, teratogenicity, mutagenicity, and carcinogenicity (Cao et al., 2022). AFs are the most frequent contaminants of peanuts, corn, wheat, rice, nuts, milk, and their by-products (Shi et al., 2018; Hajian et al., 2020). More than 10% of the global exposure to AFs was from maize, peanuts, rice, sorghum, and wheat (JECFA, 2017), which caused remarkable economic losses (Pitt and Miller, 2017). The climate change due to latitude differences and the carbon utilization pattern of *A. flavus* were the main reasons of aflatoxin contamination (Mohale et al., 2013; Van der Fels-Klerx et al., 2016). In addition, the adverse storage conditions of high humidity and high temperature caused excessive aflatoxin levels (Villers, 2014).

The biological degradation of Afs is an emerging biotechnological strategy that is considered an inexpensive and safe practice. The decontaminant activity of microorganisms is associated with fermentation processing and the binding capacity of the cell wall to the contaminant (Wochner et al., 2018). The biological degradation strategy depends on the adsorption of *Lactobacillus* (Luo et al., 2020) and probiotics (Zoghi et al., 2014) as the microbiological adsorbents and the degradation of AFs using AF oxidases, including laccases (Song et al., 2021), peroxidases (Loi et al., 2020), and lactonases (Pereyra et al., 2019). The enzymatic degradation effectively removes AF toxicity through the destruction of the furan ring and coumarin structure with the formation of nontoxic degradation products (Wang et al., 2019b). The overexpression of AF-degrading enzymes is regarded as an effective way to obtain enzymes that could be used for AF detoxification (Yang et al., 2021). However, the enzymatic separation, purification, and catalytic processes expend great cost during production (Schmidt et al., 2021). Current measures based on direct detoxification approaches, such as physical adsorption, chemical decomposition, and enzymatic degradation (Peng et al., 2018), still have great difficulties in realizing AF detoxification in the fields of food and feed (Kumar et al., 2021). Therefore, the degradation of AFB_1_ and the expression of AFB_1_-degrading enzyme during the fermentation of microorganisms would be conducive to decreasing the operating costs.

In the present study, the AFB_1_-degrading enzyme from *Trametes versicolor* (*TV-AFB_1_D*) was expressed in the recombinant *E. coli* BL21(DE3). The enzymatic characteristics and catalytic effect were investigated. In addition, *TV-AFB_1_D* was used to construct engineered *S. cerevisiae* strains by Clustered Regularly Interspaced Short Palindromic Repeats–Cas 9 (CRISPR-Cas9) technology. Two TV-AFB_1_D cassettes were integrated into the hexokinase 2 (*HXK2*) locus of *S. cerevisiae*. The degradation of AFB_1_ in AFB_1_–contaminated rice was investigated during *S. cerevisiae* fermentation. An alternative strategy for simultaneous AFB_1_ degradation and ethanol production by TV–AFB_1_D–engineered *S. cerevisiae* strains were explored using the AFB_1_-contaminated rice as the substrate.

## Materials and methods

### Plasmids, primers, and reagents

pET-28a (+) expression kit, pEASY-T1 cloning plasmid, RT-PCR kit, *E. coli* BL21(DE3), ProteinIso Ni-IDA Resin, and *E. coli* DH *5α* were from Transgen Biotech. The gel imaging system, SDS-PAGE, PCR amplification, and electrophoresis devices were manufactured by Bio-Rad Company (United States). Chirascan qCD was manufactured by Applied Photophysics Ltd. (United Kingdom). Plasmid Cas9-NAT carrying nourseothricin and ampicillin resistance genes was obtained from Addgene. The *S. cerevisiae HXK2* guide RNA (gRNA) expression vector (HXK2-gRNA) for the expression of the 20 bp gRNA was obtained through the amplification of the gRNA-Trp-Hyb plasmid (Addgene) using the designed primers in Table 1. *A. flavus* AFB_1_ was from FERMENTEK (Israel). Primer synthesis and gene sequencing were performed by Sangon Company (China). The analytically pure reagents were from Aladdin Reagent Company (China).

**Table 1:** The primers designed for gene amplification in this study.

Genes for amplification	Primers
AOX1	F-5′-gatctaacatccaaagacga-3′
	R-5′-tctcacttaatcttctgtac-3′
CaMV	F-5′-gagacttttcaaagggt-3′
	R-5′-gatctggattttagtactgg-3′
TV-AFB_1_D	F-5′-atggctcgcgcgaagtactc-3′
	R-5′-gcgcttcccaattgaggtac-3′
HXK2-gRNA	F-5′-ctcattttggaacaagtcatgttttagagctagaaatagcaag-3′
	R-5′-atgacttgttccaaaatgaggatcatttatctttcactgcgga-3′

Two pairs of primers of AOX1 and CaMV were used to amplify the expression cassettes. The primers of TV-AFB_1_D were used to amplify TV-AFB_1_D for the identification of transformants. The primers of HXK2-gRNA were used to amplify the expression vector for the transcription of guide RNA.

### Engineered *Escherichia coli* BL21(DE3) construction

In this study, *TV-AFB1D* sequences from NCBI reference sequence NW_007360323.1 was synthesized by Sangon Company (China). *TV-AFB_1_D* was inserted into a pET-28a (+) expression vector backbone by double-enzyme *Xba*I and *BamH*I digestion and integration approach. T7 promoter primer and T7 terminator primer were used to amplify the insertion sequence by sequencing. The recombinant expression vector embracing the target gene possessed the correct insertion direction and location. The downstream sequence was close to the T7 terminator primer. The confirmed expression vector was transformed into *E. coli* BL21(DE3) for recombinant expression.

### Recombinant expression of TV-AFB_1_D in *E. coli* BL21(DE3)

Isopropyl β-D-Thiogalactoside (IPTG) was used to induce the expression of the target gene in engineered *E. coli* BL21(DE3). The engineered *E. coli* BL21(DE3) was inoculated into a 100 ml Erlenmeyer flask loaded with 10 ml of LB liquid medium containing 90 μg/ml of kanamycin at 37°C with a shaking speed of 200 rpm. Different concentrations of IPTG were added to induce gene expression when cell concentration reached 0.5 OD600. After centrifugation of fermented broth at 9,000 rpm for 10 min, the supernatant and cells were obtained from the stratified solution to further detect the activity of the recombinant. The composition of fermentation broth mainly included LB liquid medium (10 mg/ml of tryptone, 5 mg/ml of yeast extract, 10 mg/ml of NaCl), 0.19 mg/ml of IPTG (0.8 mmol/l), 90 μg/ml of kanamycin, 0.5 OD600 of TV-AFB1D engineering *E. coli* BL21(DE3) cells, recombinant TV-AFB1D, and water.

### Enzymatic characteristics of recombinant TV-AFB_1_D

The conditions of 32°C and pH 7 were maintained to investigate the effect of pH value and temperature on TV-AFB_1_D activity, respectively. The optimum temperature and pH value, and kinetic parameters were determined according to the enzyme activities under the different conditions. V_max_ (mM/min) and K_m_ (mM) were calculated based on the Lineweaver–Burk method. The kinetic equation was drawn with 1/V_0_ and 1/[S] as the ordinate and abscissa, respectively. The Arrhenius plot approach was used to investigate the effect of temperature on TV-AFB_1_D stabilization by analyzing the relationship between the initial reaction speed and temperature (Fan et al., 2000).

### Secondary structure determination

The effect of temperature on the secondary structure of TV-AFB_1_D was investigated using low temperature (4°C), optimum temperature (32°C), and high temperature (70°C). Circular dichroism spectroscopy (CD) was used to investigate the effect of temperature on the secondary structure. Chirascan qCD was applied to record the CD spectrum values with 0.5 mg/ml of TV-AFB_1_D in a path length cuvette. CDPro software analyzed the data from 190 nm to 260 nm with a time-per-point of 1 s and an interval of 1 nm (Yi et al., 2019).

### TV-AFB_1_D expression cassette assembly for transformation in *Saccharomyces cerevisiae*

The TV-AFB_1_D expression cassettes assembled using the promoters of inducible aldehyde oxidase 1 (*AOX1*, NCBI Sequence ID: LT727205.1) and constitutive *Cauliflower mosaic virus* (*CaMV*) *35S* (NCBI Sequence ID: X04879.1) were synthesized according to the detailed sequences. The inducible expression cassette was assembled with the *AOX1* promoter, *TV-AFB_1_D*, and *AOX1* terminator, whereas the constitutive expression cassette was assembled using the *CaMV 35S* promoter, *TV-AFB_1_D*, and *CaMV* poly-A signal sequences. The primers for *AOX1* and *CaMV* amplification in Table 1 were used to identify the insertion gene. The primers for HXK2-gRNA amplification were used to obtain an expression vector for the transcription of a 20 bp gRNA.

### Integration of TV-AFB_1_D by CRISPR-Cas9 approach in *S. cerevisiae*

**Figure 1:**
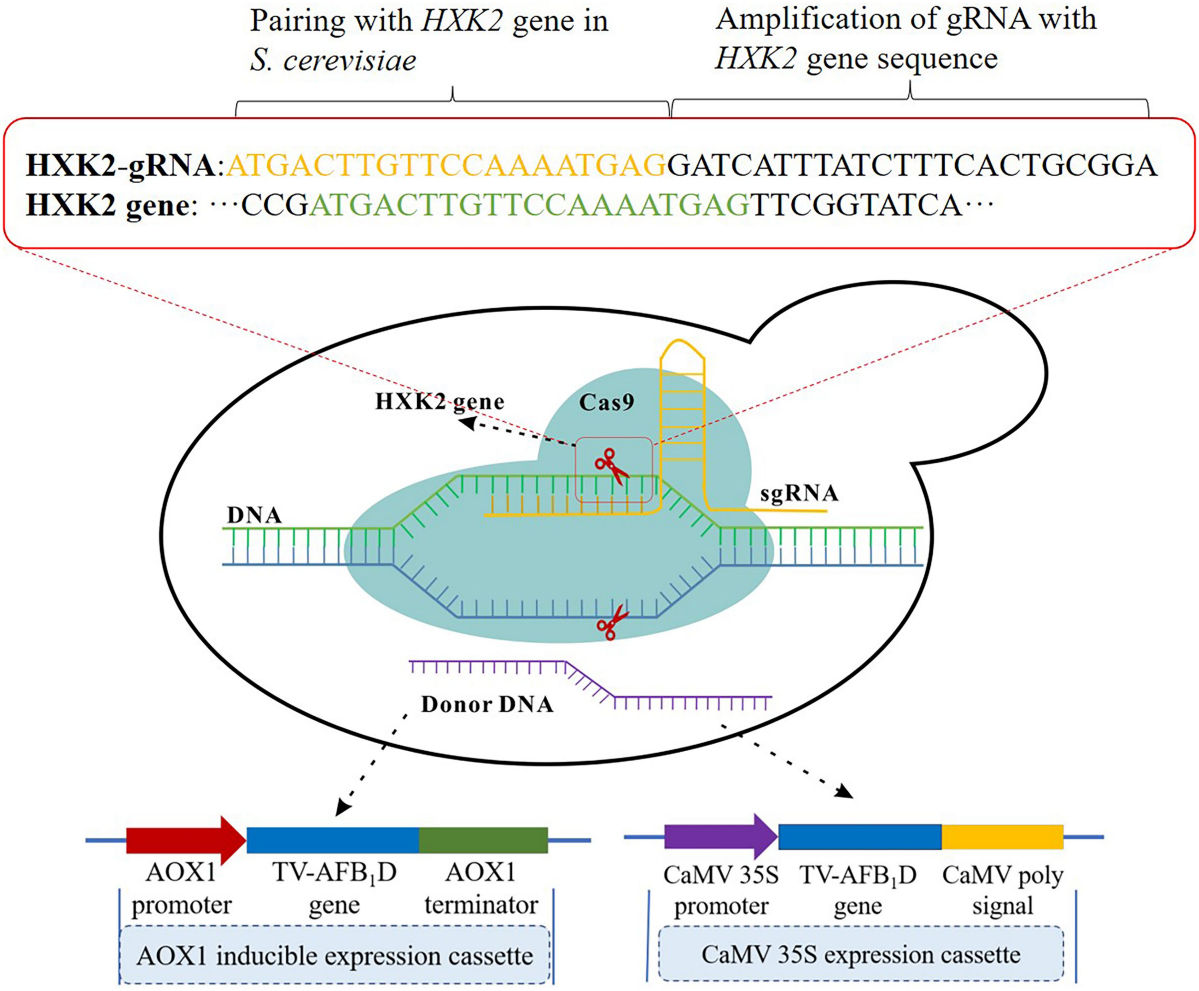
Construction strategy of TV-AFB_1_D engineered *Saccharomyces cerevisiae*. Two kinds of expression cassettes containing *AXO1* and *CaMV* promoters were assembled by the promoter, *TV-AFB_1_D*, and terminator. The integration locus of expression cassettes as donor DNA was in the *HXK2* of *S. cerevisiae* recognized by 20-bp guide RNA using the CRISPR-Cas9 technology.

The TV-AFB_1_D expression cassettes were integrated into the *S. cerevisiae* genome by *HXK2* knockout using the CRISPR-Cas9 technology (Figure 1). The nuclease expressed by the Cas9 vector was cut off by double-stranded DNA guided by gRNA. Then, the TV-AFB_1_D expression cassettes were inserted into the cut site as the donor DNA to repair the break. The TV-AFB_1_D expression cassettes were integrated into the genomic DNA of *S. cerevisiae* by the CRISPR-Cas9 approach through the following steps (Yang et al., 2018). Cas9 plasmid containing nourseothricin resistance gene was transformed into *S. cerevisiae* by the LiAc/ssDNA/PEG method (Zhang et al., 2014). The 50 μl transformation solution was sucked out and coated on the solid yeast extract peptone dextrose (YPD) medium with the components of 1% yeast extract, 2% yeast extract, and 2% glucose (w/v) containing 100 μg/ml nourseothricin. The *S. cerevisiae* transformants were used for gene integration. The HXK2-gRNA plasmid and TV-AFB_1_D expression cassettes were transformed into *S. cerevisiae* integrated with the Cas9 plasmid. The transformation solution was incubated at 30°C on a solid YPD medium containing 100 μg/ml nourseothricin and 300 μg/ml hygromycin B for 48 h, and the putative transformants were screened out for further identification.

### Identification and recombinant expression of TV-AFB_1_D in *S. cerevisiae*

The putative *S. cerevisiae* transformants were incubated in a YPD medium at 30°C with a shaking speed of 200 rpm. The primers for *TV-AFB_1_D* were used to amplify the genomic DNA of the *S. cerevisiae* transformants. After confirmation, a profile analysis of the recombinant TV-AFB_1_D was performed by sodium dodecyl sulfate-polyacrylamide gel electrophoresis (SDS–PAGE). The recombinant TV-AFB_1_D of the engineered *S. cerevisiae* in the supernatant was concentrated through an ultrafiltration membrane (Singh et al., 2020). The *S. cerevisiae* transformants after confirmation were inoculated into 500 ml triangular flasks with 200 ml of YPD medium with a shaking speed of 200 rpm at 30°C. The recombinant TV-AFB_1_D of the *AOX1*-engineered *S. cerevisiae* was induced after the addition of methanol to a final concentration of 0.8% (v/v). The supernatant was collected after centrifugation at 7,500 rpm for 20 min.

### Cell proliferation of engineered *S. cerevisiae*

The cell proliferation of *S. cerevisiae* was investigated in a YPD medium at 30°C with a shaking speed of 200 rpm. *S. cerevisiae* concentration was determined using the absorbance value under the wavelength of 600 nm. When the OD600 value reached 1, 1 ml of broth was sucked out and poured into a 250 ml triangular bottle containing 100 ml of YPD medium. The effect of gene knockout on the growth of engineered *S. cerevisiae* was investigated according to the OD600 values (Oh and Jin, 2020).

### Determination of glucose and ethanol concentrations

The supernatant of the fermentation broth was obtained after centrifugation at 10,000 rpm for 10 min. Trichloroacetic acid (10% v/v) was added to the supernatant in equal proportion for protein removal after treatment at 4°C for 12 h. The supernatant was further purified by filtration through a 0.45 μm filter membrane. Glucose and ethanol concentrations were determined by high-performance liquid chromatography (HPLC) using a Waters series HPLC system equipped with a refractive detector and SUGAR SH1011 column, a mobile phase of 0.01 mol/l H_2_SO_4_, a flow rate of 0.8 ml/min, and a column temperature of 50°C.

### Saccharification, fermentation, and *in situ* degradation of AFB_1_

The saccharification of rice power was carried out by the double-enzyme method. The starch of rice was hydrolyzed by the mixture of 20 g rice power, 100 ml of water, and 0.02 g α-amylase (40 U/mg) at 70°C for 30 min. After the pH value was adjusted to 4.5, 0.08 g glucoamylase (50 U/mg) was added for the release of glucose at 60°C for 4 h. The saccharified liquid was used to prepare the fermentation broth containing 1% yeast extract (w/v) and 2% peptone (w/v). The two kinds of engineered *S. cerevisiae* strains were inoculated into the fermentation broth with a shaking speed of 200 rpm at 30°C. In the fermentation processing of the inducible *AOX1-*engineered *S. cerevisiae*, 0.8% methanol (v/v) was added for the induction of TV-AFB_1_D.

### AFB_1_ extraction and HPLC determination

AFB_1_ extraction was performed by batch micro-solid phase extraction (Chmangui et al., 2021). The rice powder (5 g) was soaked in a 50 ml tube containing an 84% acetonitrile solution (v/v). The supernatant was collected by centrifugation at 6,000 rpm for 10 min. Fat, protein, pigment, and carbohydrate in the extraction solution were removed by a solid-phase column for AF purification (Zhang et al., 2020). AFB_1_ detection was performed by the HPLC method using the following parameters: a mobile phase of 4:6 methanol–water, C18 reversed-phase column with a UV detector, a detection wavelength of 365 nm, and a flow rate of 0.6 ml/min. AFB_1_ content was determined by the external standard method (Munoz-Solano and Gonzalez-Penas, 2020).

### Data analysis

All the data were presented in the form of mean ± standard deviation with three replicates. Statistical analyses were performed by Origin 9 Software.

## Results

### Expression of recombinant TV-AFB_1_D in *E. coli* BL21(DE3)

**Figure 2:**
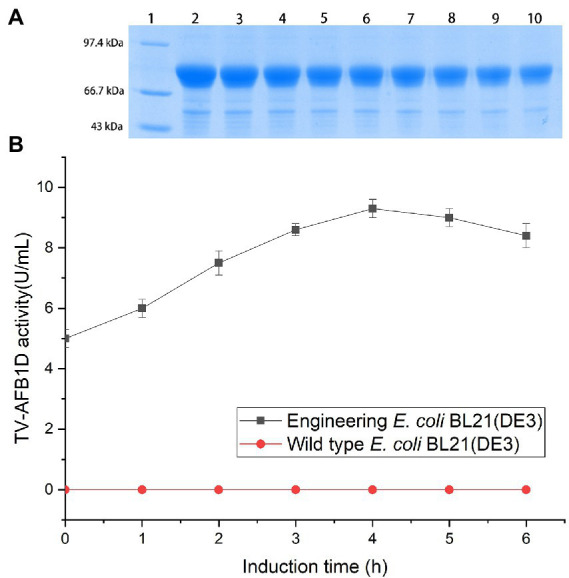
Expression of TV-AFB_1_D in *E. coli* BL21(DE3) by IPTG induction **(A)** Profile of TV-AFB_1_D expressed in engineered *E. coli* BL21(DE3) induced by IPTG *via* SDS-PAGE approach. Note: Lane 1 indicated protein marker; Lane 2–10 indicated the recombinant TV-AFB_1_D in engineered *E. coli* BL21(DE3) induced by 1.4, 1.2, 1, 0.8, 0.6, 0.4, 0.3, 0.2, 0.1 mmol/l, respectively. **(B)** Expression of TV-AFB_1_D in the transformant and wild-type *E. coli* BL21(DE3).

**Figure 3:**
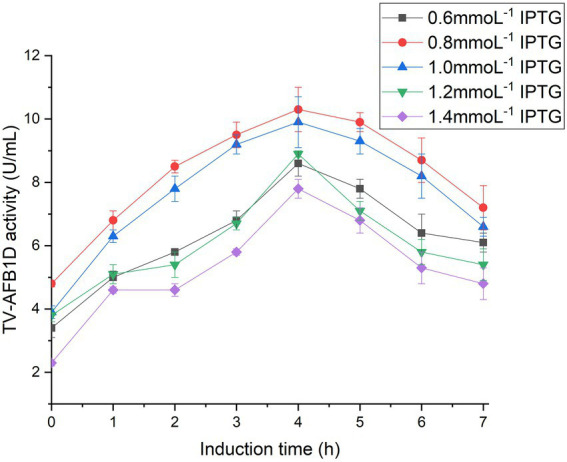
Inducible expression of TV-AFB_1_D by IPTG concentrations and induction time.

*TV-AFB_1_D* was integrated into a pET-28a (+) expression plasmid. The expression vector was confirmed by sequencing. The correct pET-28a (+) vector embracing *TV-AFB_1_D* was transformed into *E. coli* BL21(DE3). The size of TV-AFB_1_D expressed in engineered *E. coli* BL21(DE3) was appropriately 77 kDa in the presence of different concentrations of IPTG (Figure 2A). The expression of engineered *E. coli* BL21(DE3) was induced in the presence of IPTG. The concentration of 0.8 mmol/l of IPTG could effectively induce the expression of TV-AFB_1_D in engineered *E. coli* BL21(DE3). Thus, the expression of the recombinant TV-AFB_1_D in the engineered *E. coli* BL21(DE3) at 37°C was investigated after the addition of 0.8 mmol/l of IPTG. The highest activity of TV-AFB_1_D reached 9.3 U/ml for 4 h of induction (Figure 2B). As the control, the wild-type *E. coli* BL21(DE3) could not produce any TV-AFB_1_D activity. IPTG concentration and induction time were used to investigate TV-AFB_1_D activity under the same induction conditions (Figure 3). The combined parameters of 0.8 mmol/l of IPTG and 4 h induction led to the highest TV-AFB_1_D activity among the set combinations of 0.6–1.4 mmol/l of IPTG and 0–7 h induction time.

### Effect of temperature and pH values on TV-AFB_1_D activity

**Figure 4:**
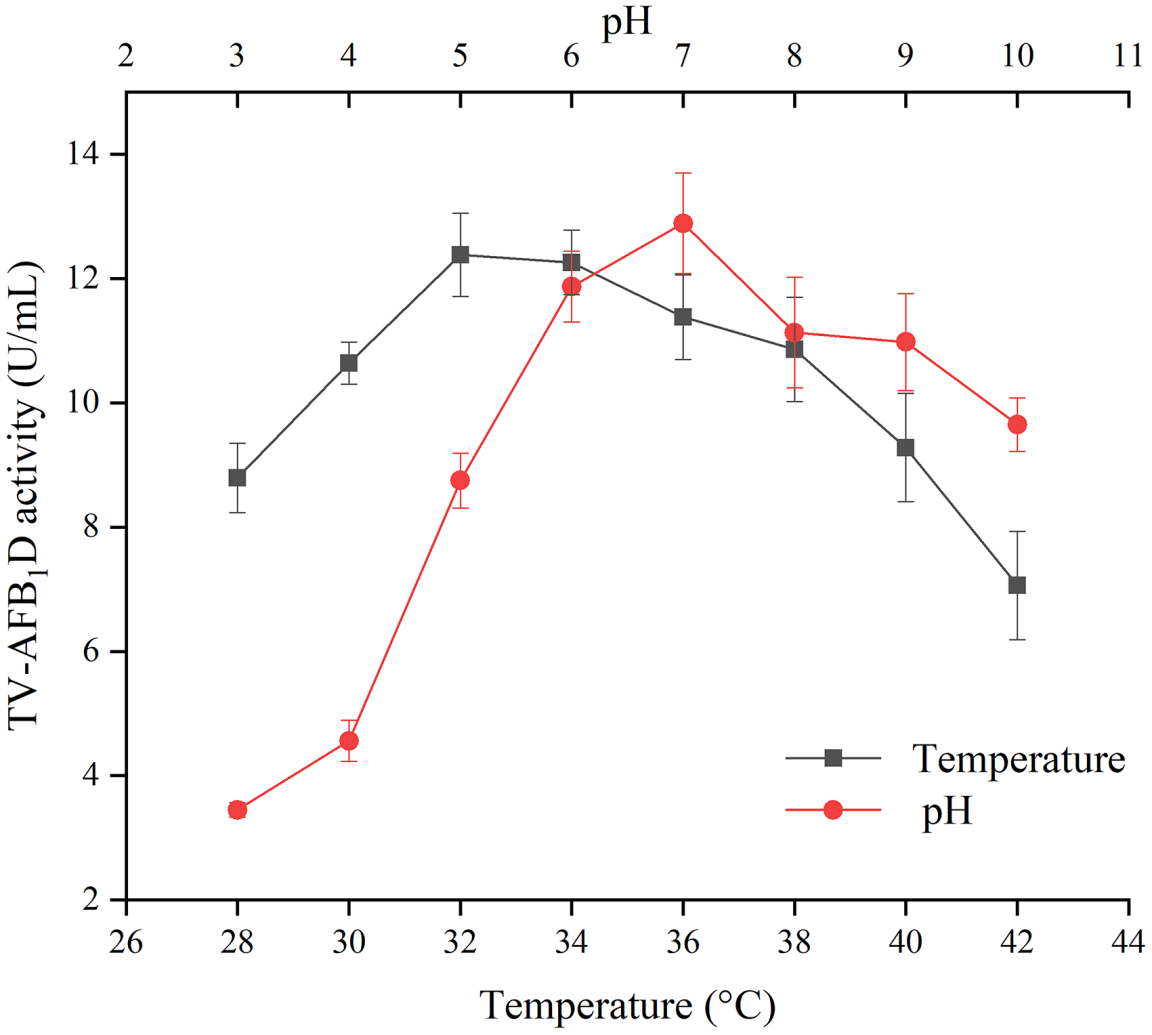
Effect of temperature and pH on TV-AFB_1_D activity.

The effects of temperature and pH values on the activity of recombinant TV-AFB_1_D were investigated to optimize the catalytic conditions (Figure 4). Both temperature and pH values resulted in the changes of TV-AFB_1_D activity. The activity of recombinant TV-AFB_1_D reached 12.38 U/ml at 32°C, which was the highest among those during the temperatures of 28–42°C. In addition, the activity of TV-AFB_1_D was 12.89 U/ml when pH value was adjusted to 7, which was the highest among those at pH values of 3–10. Too high and low temperature and pH values would decrease the activity of recombinant TV-AFB_1_D. Therefore, TV-AFB_1_D had characteristics of the optimal temperature of 32°C and pH value of 7.

### Determination of kinetic parameters

**Figure 5:**
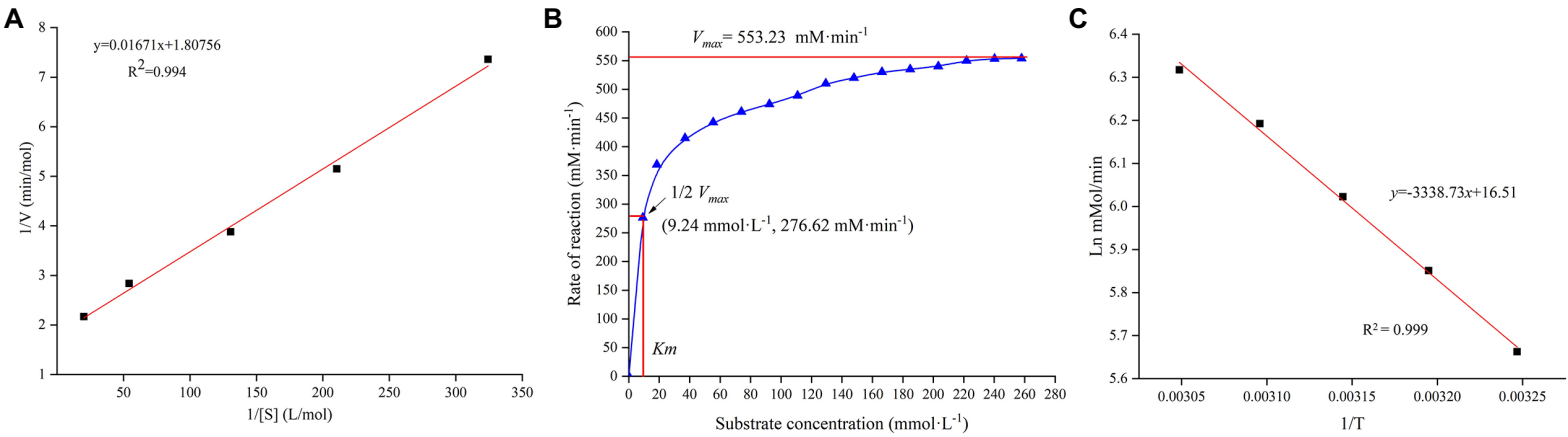
Enzymatic characteristics of recombinant TV-AFB_1_D. **(A)** The kinetic equation of TV-AFB_1_D drawn by Lineweaver–Burk equation based on the double reciprocal of substrate concentration and catalytic rate; **(B)** Correlation between substrate concentration and reaction rate of TV-AFB_1_D; **(C)** Arrhenius plot equation of TV-AFB_1_D based on the reciprocal of temperature.

The multiple kinetic parameters of TV-AFB_1_D were determined according to their specific calculation formulas in Figure 5. The Lineweaver–Burk equation was used to draw the kinetic equations of TV-AFB_1_D with a formula of *y* = 0.01671*x* + 1.80756 (*R*^2^ = 0.994; Figure 5A). In addition, other parameters of *K_m_* = 9.24 mM, *V_max_* = 553.23 mM/min, *Kcat* = 0.07392 (s^−1^), and *Kcat*/*K_m_* = 8 M^−1^ s^−1^ were calculated according to the relation between substrate concentrations and reaction rate (Figure 5B). Further, Arrhenius plot method was used to determine the relationship between the initial catalytic speed and reaction temperature of TV-AFB_1_D with a formula of ln *v* = 16.51–3338.73/T (*R*^2^ = 0.999;. Figure 5C).

### Effect of temperature on the secondary structure

**Figure 6:**
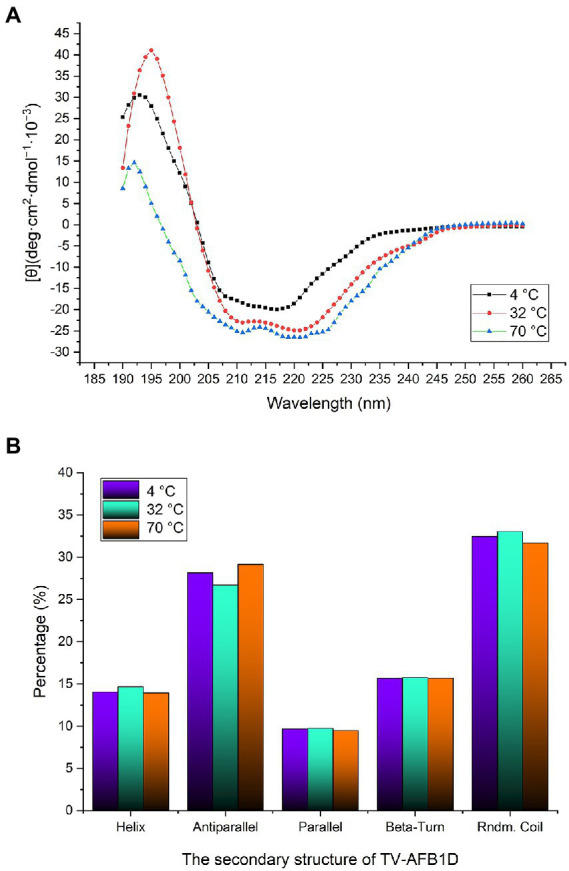
Determination of TV-AFB1D secondary structure. **(A)** CD spectrum method determined TV-AFB_1_D secondary structure; **(B)** Types and percentages of the secondary structure of TV-AFB_1_D at 4, 32 and 70°C.

The CD spectrum method was used to analyze the secondary structure of TV-AFB_1_D at 4°C, 32°C, and 70°C (Figure 6A). At 32°C, the highest peak value [θ] was 41.01 deg.·cm^2^·dmol^−1^·10^−3^, which was 1.34-fold at 4°C (30.52 deg.·cm^2^·dmol^−1^·10^−3^) and 2.82-fold at 70°C (14.56 deg.·cm^2^·dmol^−1^·10^−3^). The types of the secondary structure are calculated in Figure 6B. The percentages of random coil and helix at 32°C were highest among the temperatures. The percentage of antiparallel structure at 32°C was lower than those at 4°C and 70°C. Three different treatment temperatures led to similar percentages of parallel and beta-turn structures. Therefore, the percentages of the random coil, helix, and antiparallel structures could be the main factors affecting the activity and stability of TV-AFB_1_D.

### Effect of treatment time on the concentration of AFB_1_

**Figure 7:**
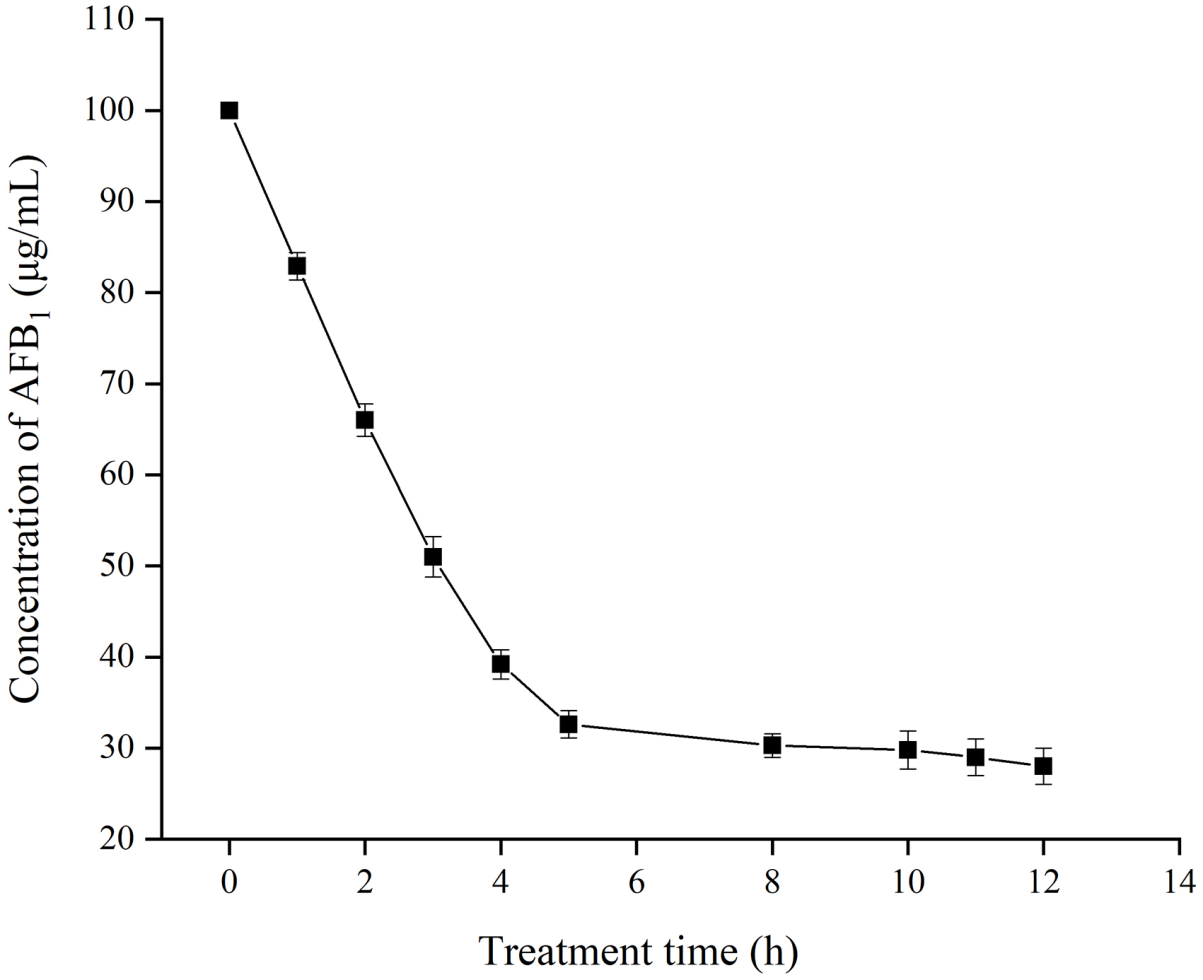
Effect of time on the residual concentration of AFB_1_ by recombinant TV-AFB_1_D.

The effect of treatment time on the residual concentration of AFB_1_ was investigated in the presence of TV-AFB_1_D (Figure 7). The content of AFB_1_ gradually decreased from 100 μg/ml to 32.6 μg/ml after catabolism for 5 h. Then, the decrease rate of AFB_1_ content substantially slowed down with the next treatment of 5–12 h. Therefore, 67.4% of AFB_1_ was eliminated by TV-AFB_1_D after 5 h of catabolism.

### Transformation of TV-AFB_1_D expression cassettes into *S. cerevisiae*

**Figure 8:**
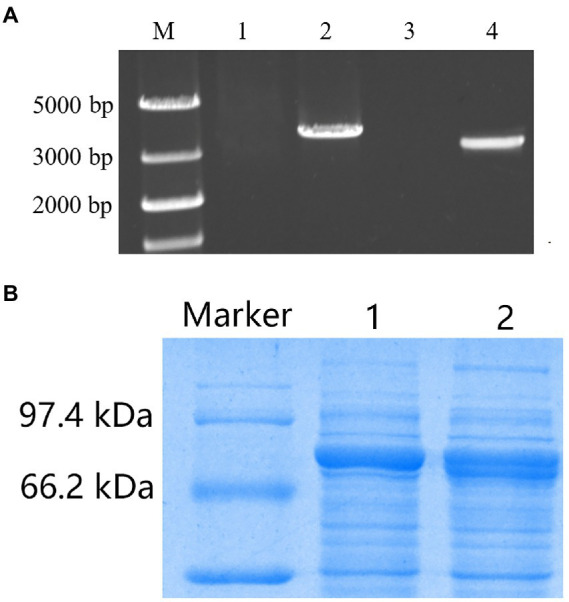
Recombinant expression of TV-AFB_1_D in *Saccharomyces cerevisiae*. **(A)** Amplification of two kinds of TV-AFB_1_D expression cassettes. Lanes 1 and 2 represented the control and *AOX1* expression cassette with a size of 3,663 bp, respectively; lanes 3 and 4 represented the control and *CaMV* expression cassette with a size of 3,048 bp, respectively; **(B)** Recombinant TV-AFB_1_D by the SDS-PAGE approach. Lanes 1 and 2 represented *AXO1-* and *CaMV-*engineered strains of *S. cerevisiae*, respectively.

*AOX1* inducible and *CaMV* constitutive expression cassettes have sizes of 3,663 and 3,048 bp, respectively (Figure 8A). After sequencing confirmation, the two expression cassettes were transformed into wild-type *S. cerevisiae* on the screening medium containing nourseothricin and hygromycin B. The primers designed for TV-AFB_1_D sequences were used to amplify the genomic DNA of the putative transformants for transformant identification. The sequences of the isolated DNA were further confirmed by the sequencing method. The engineered strains were constructed by integrating the two TV-AFB_1_D expression cassettes into the *S. cerevisiae* genome.

### Profile analysis of recombinant TV-AFB_1_D by SDS–PAGE

SDS–PAGE was used to analyze the profiles of recombinant TV-AFB_1_D (Figure 8B). The recombinant TV-AFB_1_D was expressed by the two kinds of engineered *S. cerevisiae* with a size of approximately 77 kDa. In addition, the wild-type *S. cerevisiae* could not express recombinant TV-AFB_1_D. Thus, engineered *S. cerevisiae* could express the recombinant TV-AFB_1_D under the control of *AOX1* and *CaMV* promoters.

### Detection of AFB_1_ catabolism by recombinant TV-AFB_1_D

**Figure 9:**
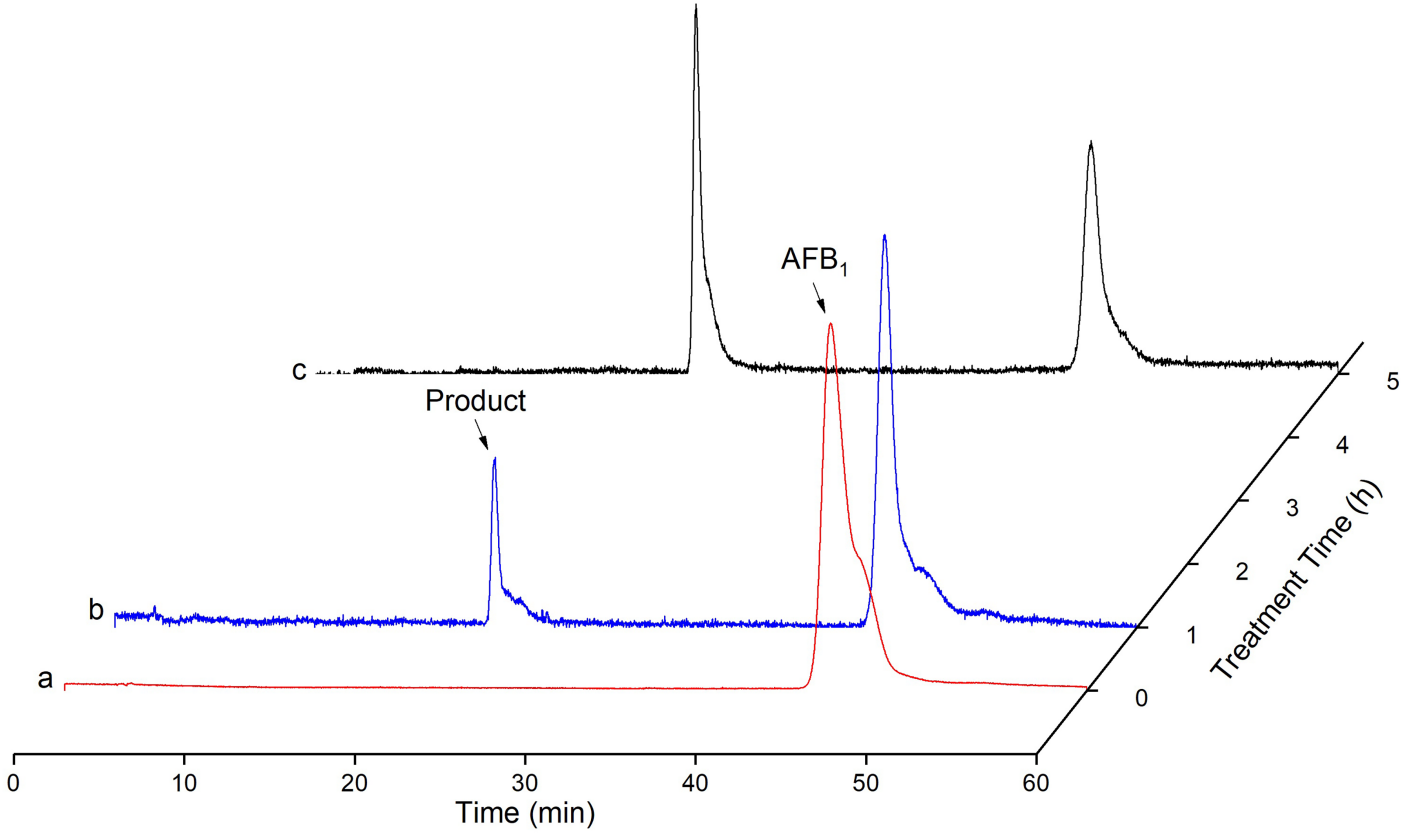
Determination of AFB_1_ degraded by TV-AFB_1_D using HPLC approach. a, b, and c, respectively, represented HPLC standard AFB_1_, products after catabolism for 2 h and 4 h.

The HPLC method was used to detect AFB_1_ and its catabolites by recombinant TV-AFB_1_D (Figure 9). AFB_1_ possessed a chromatographic peak after the run time of 45 min. The catabolites of AFB_1_ by recombinant TV-AFB_1_D were also detected by HPLC with a run time of 22 min. The result indicated that a new product was formed under the catabolism of recombinant TV-AFB_1_D.

### *HXK2* knockout affecting the growth, ethanol production, and glucose consumption of *S. cerevisiae*

**Figure 10:**
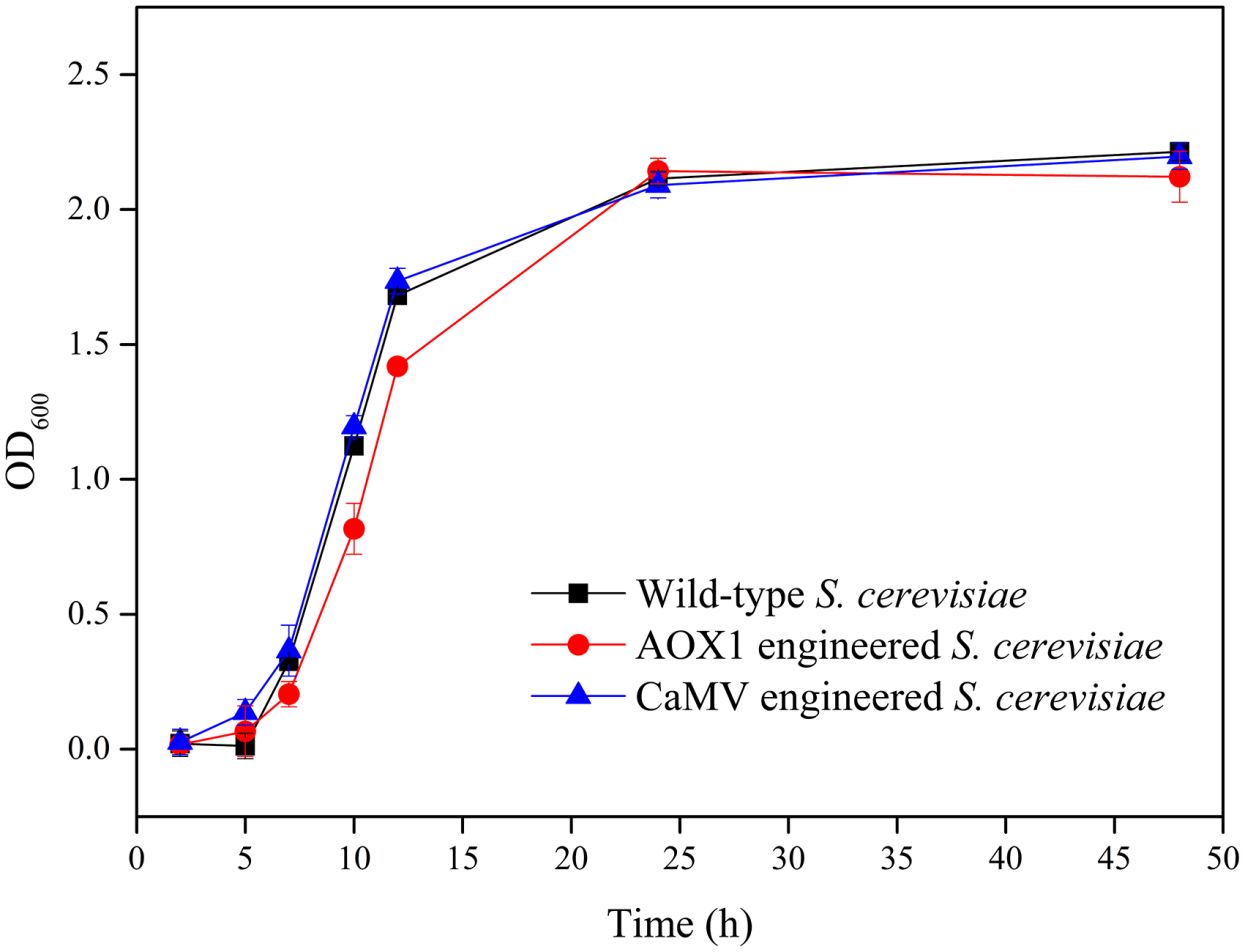
Cell growth of AXO1 and CaMV engineered strains of *S. cerevisiae.*

The absorbance of the fermentation broth at the wavelength of 600 nm was determined to investigate the effect of the gene integration of TV-AFB_1_D expression cassettes on the growth of *S. cerevisiae* mutants with *HXK2* knockout (Figure 10). The results showed that *S. cerevisiae* mutants integrated with *AOX1* and *CaMV* expression cassettes had similar growth curves as the wild-type *S. cerevisiae* during fermentation for 48 h. Therefore, the *HXK2* mutation of *S. cerevisiae* by gene knockout did not affect the cell proliferation of the engineered strains.

**Figure 11:**
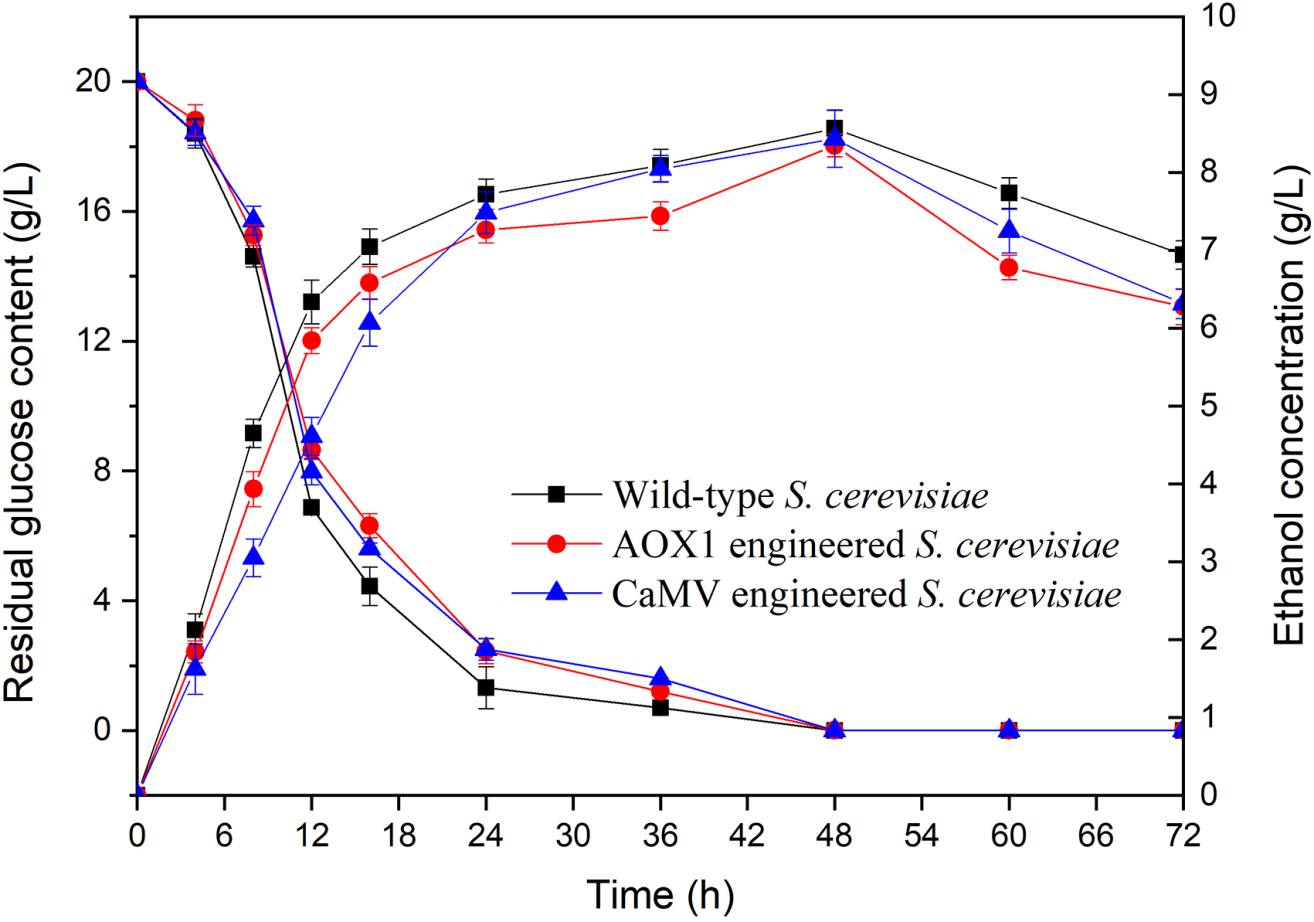
Effect of fermentation time on glucose consumption and ethanol production.

The residual glucose contents and ethanol concentrations of the engineered and wild-type *S. cerevisiae* strains were determined during fermentation for 72 h (Figure 11). During the initial fermentation (0–24 h), all the strains had a rapid increase in ethanol concentration from 0 g/l to 7.5 g/l. All the strains reached the highest ethanol concentrations after fermentation for 48 h. The ethanol concentrations from engineered *S. cerevisiae* strains carrying *AOX1* (8.35 g/l) and *CaMV* (8.43 g/l) promoters were 97.4 and 98.4% of that in the wild-type strain (8.57 g/l), respectively. The integration of TV-AFB_1_D expression cassettes did not affect ethanol production during fermentation.

Residual glucose contents were investigated during the fermentation of the wild-type and engineered *S. cerevisiae* strains for 72 h (Figure 11). The results indicated that the two kinds of engineered *S. cerevisiae* strains had similar trends in glucose content as that of the wild-type *S. cerevisiae*. The residual glucose contents markedly decreased during fermentation time at 0–24 h. In addition, the residual glucose contents slightly decreased during the subsequent fermentation at 24–48 h. Almost all the glucose was consumed by the tested strains during fermentation at 48–72 h. The integration of TV-AFB_1_D expression cassettes by *HXK2* knockout did not affect the glucose consumption of the engineered *S. cerevisiae* strains.

### *In situ* catabolism of AFB_1_ and fermentation of AFB_1_-contaminated rice

**Figure 12:**
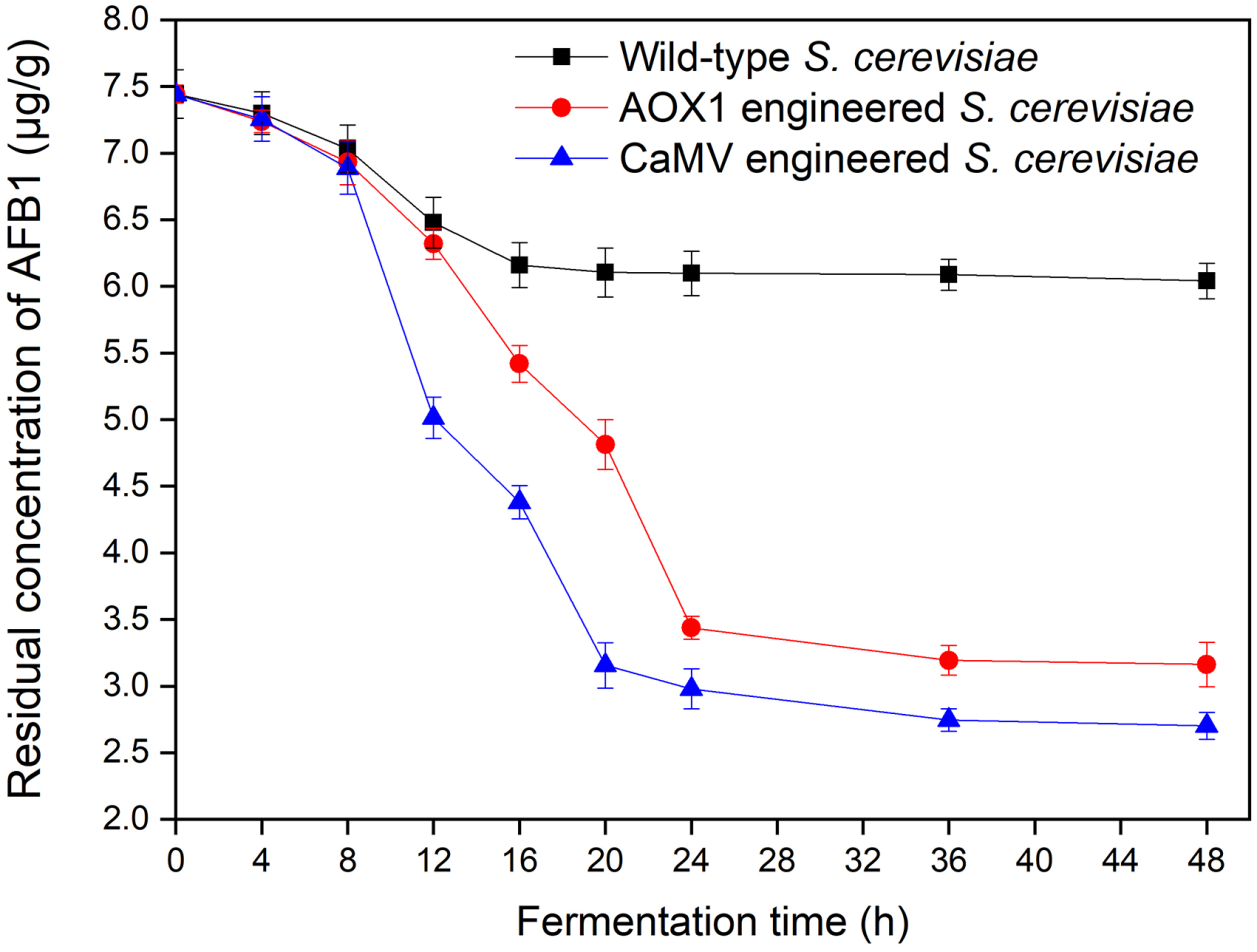
Concentrations of AFB_1_ during the fermentation from AFB_1_-contaminated rice.

The starch of AFB_1_-contaminated rice was converted into glucose under the catabolism of α-amylase and glucoamylase. The saccharification solution was used to prepare the liquid broth for ethanol production by *S. cerevisiae* fermentation. The residual AFB_1_ concentrations of *S. cerevisiae* in the fermentation broth were investigated during the fermentation for 48 h (Figure 12). The results indicated that the two kinds of engineered strains possessed higher AFB_1_ degradation efficiencies than the wild-type *S. cerevisiae*. The concentrations of residual AFB_1_ markedly decreased during the initial fermentation time of 0–24 h and slightly decrease at 24–48 h of fermentation. The concentrations of residual AFB_1_ from the wild-type strain (6.1 μg/g), *AOX1*-engineered strain (3.4 μg/g), and *CaMV*-engineered strain (2.9 μg/g) after 24 h of fermentation were lower than the initial AFB_1_ concentration of 7.4 μg/g. The *AOX1-* and *CaMV*-engineered *S. cerevisiae* strain resulted in the degradation of 54 and 61% of AFB_1_, respectively, which were higher than the wild-type *S. cerevisiae* (18% AFB_1_). Thus, both engineered strains could degrade AFB_1_ during the fermentation of the saccharification solution from AFB_1_-contaminated rice for ethanol production.

## Discussion

AFB_1_ widely exists in nature and even breaks out in certain environments as a chemically stable mycotoxin. It threatens food and animal feed security and causes an economic challenge and health hazard to consumers. Enzymatic hydrolysis is an effective detoxification approach of AFB_1_ owing to its nutrition maintenance without toxicity residue. The specific enzymes can directly degrade aflatoxins without the shortcoming of the application of a whole microorganism. The F_420_H_2_-dependent reductases reduced the α,β-unsaturated ester and destabilized the lactone ring (Taylor et al., 2010). Various enzymes such as peroxidases (Yehia, 2014), laccases (Alberts et al., 2009), oxidases (Wu et al., 2015), and reductases (Taylor et al., 2010) have been identified as responsible for AFB_1_ degradation (Table 2). The optimum temperature of 32°C and pH value of 7 could maintain better enzymic activity and stability of TV-AFB_1_D compared with other enzymes. The previously reported enzymes from different host bacteria demonstrate the optimum temperature of 20–70°C and pH values of 4.5–10 (Table 2). The optimum temperature and pH of AFB_1_ degradation enzyme are identical to the fermentation conditions of *S. cerevisiae* (32°C and pH 7). This coincidence can facilitate yeast fermentation and AFB_1_ degradation by TV-AFB_1_D. In addition, TV-AFB_1_D can convert 67.4% of AFB_1_ into other compounds in 5 h, whereas most of the other enzymes reached a high conversion rate after processing for 48–72 h. Therefore, the recombinant TV-AFB_1_D has good application value for its high catalytic efficiency.

**Table 2:** Enzymatic detoxification of AFB_1_ and biotransformation.

Microbial sources and enzymes	Optimum temperature, pH, molecular weights	Initial AFB_1_ concentrations	Conversion efficiency (time)
*M. smegmatis* AFB reductase (Li et al., 2019)	22°C, pH 7.4, 32.4 kDa	3.12 μg/ml	63% (8 h)
*M. fulvus* AFB degradation enzyme (Zhao et al., 2011)	35°C, pH 6, 32 kDa	0.1 μg/ml	71.89% (48 h)
*Armillariella tabescens* AFB oxidase (Wu et al., 2015)	35°C, pH 6.8, 51.8 kDa	3.12 μg/ml	**/**
*Phanerochaete sordida* peroxidase (Wang et al., 2011)	30°C, pH 4.5	50 μg/ml	86% (24 h)
*Pleurotus ostreatus* peroxidase (Yehia, 2014)	25°C, pH 4-5, 42 kDa	312 μg/ml	90% (48 h)
*Bacillus subtilis* laccase [35; 40] (Alberts et al., 2009; Wang et al., 2019a)	50°C, pH 6–10, 65 kDa	1.4 μg/ml	98%
*Pleurotus pulmonarius* laccase (Loi et al., 2016)	25°C, 35 kDa	/	90% (72 h)
*Armoracia rusticana* peroxidase [42; 43] (Mishra and Das, 2003; Sibaja et al., 2019)	20°C, pH 6	10 μg/ml	60% (1 h)
*B. shackletonii* AFB-degrading enzyme (Xu et al., 2017)	70°C, pH 8, 22 kDa	0.1 μg/ml	47.51% (72 h)
*P. aeruginosa* AFB-degrading enzyme (Song et al., 2019)	65°C, pH 6, 48 kDa	2.5 μg/ml	65.6% (72 h)
*T. versicolor* AFB_1_-degrading enzyme, this study	32°C, pH 7, 77 kDa	100 μg/ml	67.4% (5 h)

**Figure 13:**
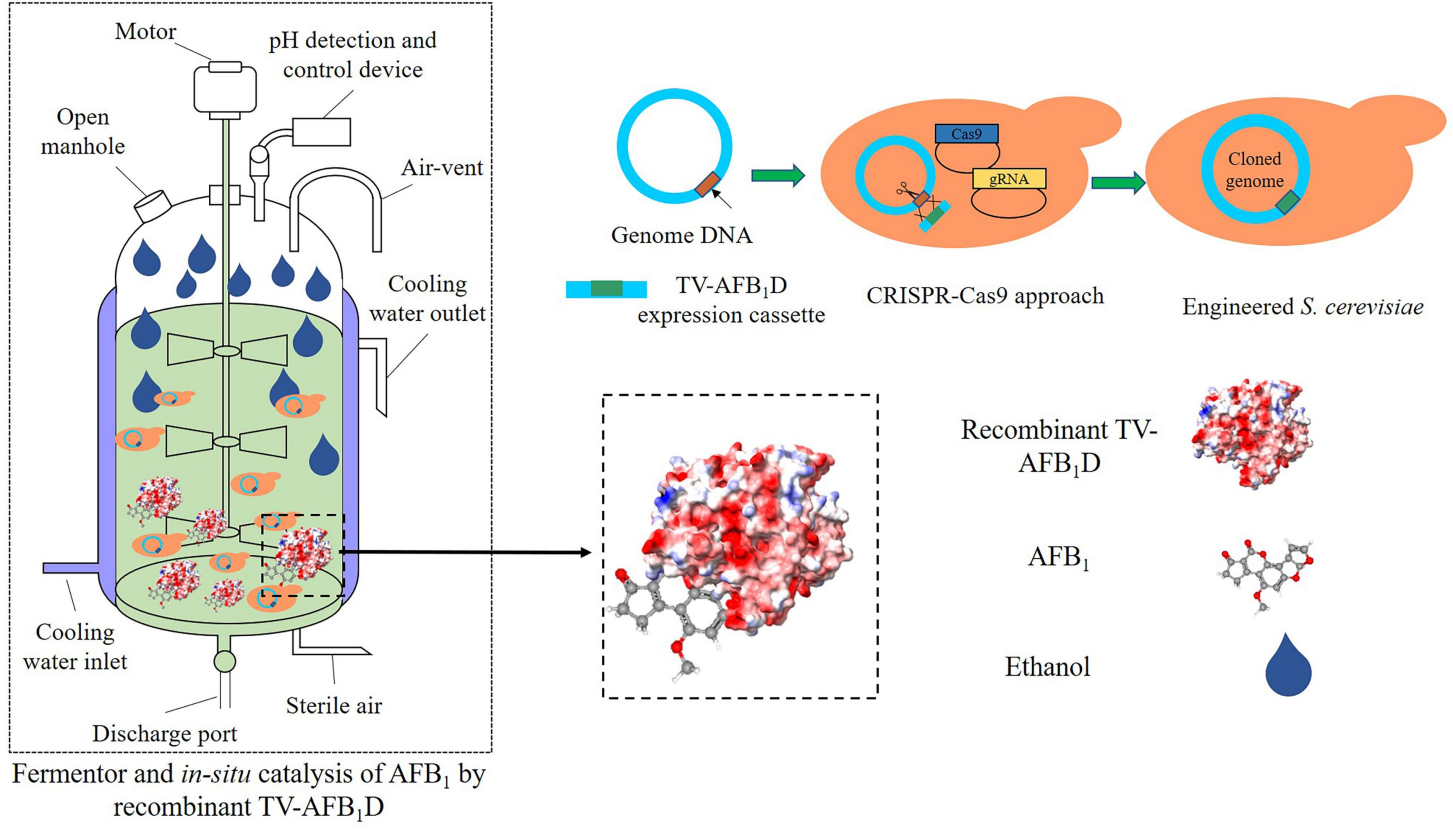
Strategy for AFB_1_ degradation and ethanol production by TV-AFB_1_D-engineered *S. cerevisiae* using AFB_1_-contaminated rice as material.

The cell wall of yeast as mycotoxin adsorbents could reduce the exposure of animals to mycotoxins to some extent (Yiannikouris et al., 2021). However, the adsorption capacity is affected by the composition, thickness, mannan-oligosaccharide, and β-glucan content of the cell walls of yeast (Pereyra et al., 2018). In the present study, we demonstrated the expression of *TV-AFB_1_D* and *in situ* AFB_1_ degradation during the ethanol fermentation of engineered *S. cerevisiae* strains. The TV-AFB_1_D-engineered *S. cerevisiae* strains are capable of AFB_1_ degradation during the fermentation of AFB_1_-contaminated rice for ethanol production without the addition of any other detoxification agents. Based on the design concept and study result, we proposed a new strategy for the AFB_1_ degradation of AFB_1_-contaminated rice by TV-AFB_1_D-engineered *S. cerevisiae* strains during the fermentation for ethanol production (Figure 13). In this strategy, the engineered *S. cerevisiae* strains integrated with TV-AFB_1_D expression cassettes were constructed by CRISPR-Cas9 knockout. The gene integration in the *HXK2* locus did not markedly affect the growth and ethanol production of the engineered strains. The application of this strategy in AFB_1_-contaminated grain could effectively decrease the AFB_1_ concentration during fermentation without the addition of extra enzymes, microorganisms, and adsorbents. Therefore, the *TV-AFB_1_D*-engineered *S. cerevisiae* strains could degrade about 60% of AFB_1_ from AFB_1_-contaminated rice by *in situ* AFB_1_ degradation. TV-AFB_1_D has application potential in the AFB_1_ degradation from contaminated agricultural products.

## Conclusion

The recombinant TV-AFB_1_D with a size of appropriately 77 KDa expressed in engineered *E. coli* BL21(DE3) and *S. cerevisiae*. The kinetic equation of TV-AFB_1_D was *y* = 0.01671*x* + 1.80756. The concentration of AFB_1_ from contaminated rice decreased from the initial 100 μg/ml to the final 32.6 μg/ml after catabolism for 5 h in the presence of TV-AFB_1_D. After fermentation for 24 h, the AFB_1_ contents of the wild-type, *AOX1*-engineered, and *CaMV*-engineered *S. cerevisiae* strains were 6.1, 3.4, and 2.9 μg/g from the initial concentration of 7.4 μg/g, respectively. In addition, an alternative strategy was proposed to degrade the AFB_1_ from AFB_1_-contaminated grain using TV-AFB_1_D-engineered *S. cerevisiae* strains during the fermentation processing. This measure has various advantages of reducing the steps of detoxification treatment, reducing production costs, and ensuring the safety of downstream products. Therefore, TV-AFB_1_D-engineered *S. cerevisiae* has an important development value to produce ethanol by simultaneous detoxification and fermentation with mycotoxin-contaminated crops as substrates.

## Data availability statement

The raw data supporting the conclusions of this article will be made available by the authors, without undue reservation.

## Author contributions

PY provided conceptualization and performed writing. SL performed the experiment. ShuJ and SuJ provided software. ZZ and ShtJ provided resources. WX, JC, and WW performed investigation. All authors contributed to the article and approved the submitted version.

## Funding

This research was funded by the Natural Science Foundation of Anhui Province (1908085MC80) and Major Science and Technology Projects of Anhui Province (202003c08020001 and 202103b06020014).

## Conflict of interest

The authors declare that the research was conducted in the absence of any commercial or financial relationships that could be construed as a potential conflict of interest.

## Publisher’s note

All claims expressed in this article are solely those of the authors and do not necessarily represent those of their affiliated organizations, or those of the publisher, the editors and the reviewers. Any product that may be evaluated in this article, or claim that may be made by its manufacturer, is not guaranteed or endorsed by the publisher.
